# Vitamin D Status Increases During Pregnancy and in Response to Vitamin D Supplementation in Rural Gambian Women

**DOI:** 10.1093/jn/nxz290

**Published:** 2019-12-13

**Authors:** Kerry S Jones, Sarah R Meadows, Inez Schoenmakers, Ann Prentice, Sophie E Moore

**Affiliations:** 1 MRC Nutrition and Bone Health Research Group, MRC Elsie Widdowson Laboratory, Cambridge, UK; 2 NIHR Nutritional Biomarker Laboratory, MRC Epidemiology Unit, University of Cambridge, Cambridge, UK; 3 Norwich Medical School, University of East Anglia, Norwich, UK; 4 MRC Unit The Gambia at the London School of Hygiene and Tropical Medicine, Fajara, The Gambia; 5 Department of Women and Children's Health, King's College London, UK

**Keywords:** Africa, nutritional requirements, pregnancy, vitamin D metabolism, cholecalciferol, season, supplementation, lipid-based nutrient supplement

## Abstract

**BACKGROUND:**

Vitamin D is important to maternal, fetal, and infant health, but quality data on vitamin D status in low- and middle-income countries and response to cholecalciferol supplementation in pregnancy are sparse.

**OBJECTIVE:**

We characterized vitamin D status and vitamin D metabolite change across pregnancy and in response to cholecalciferol supplementation in rural Gambia.

**METHODS:**

This study was a secondary analysis of samples collected in a 4-arm trial of maternal nutritional supplementation [iron folic acid (FeFol); multiple micronutrients (MMN); protein energy (PE) as lipid-based supplement; PE + MMN]; MMN included 10 μg/d cholecalciferol. Plasma 25-hydroxycholecalciferol [25(OH)D_3_], 24,25-dihydroxycholecalciferol [24,25(OH)_2_D_3_], and C3-epimer-25-hydroxycholecalciferol [3-epi-25(OH)D_3_] were measured by LC-MS/MS in 863 women [aged 30 ± 7 y (mean ± SD)] in early pregnancy (presupplementation) and late pregnancy, (gestational age 14 ± 3 and 30 ± 1 wk). Changes in 25(OH)D_3_ and vitamin D metabolite concentrations and associations with pregnancy stage and maternal age and anthropometry were tested.

**RESULTS:**

Early pregnancy 25(OH)D_3_ concentration was 70 ± 15 nmol/L and increased according to pregnancy stage (82 ± 18 and 87 ± 17 nmol/L in the FeFol and PE-arms) and to cholecalciferol supplementation (95 ± 19 and 90 ± 20 nmol/L in the MMN and PE + MMN-arms) (*P* < 0.0001). There was no difference between supplemented groups. Early pregnancy 25(OH)D_3_ was positively associated with maternal age and gestational age. Change in 25(OH)D_3_ was negatively associated with late pregnancy, but not early pregnancy, triceps skinfold thickness. The pattern of change of 24,25(OH)_2_D_3_ mirrored that of 25(OH)D_3_ and appeared to flatten as pregnancy progressed, whereas 3-epi-25(OH)D_3_ concentration increased across pregnancy.

**CONCLUSION:**

This study provides important data on the vitamin D status of a large cohort of healthy pregnant women in rural Africa. Without supplementation, vitamin D status increased during pregnancy, demonstrating that pregnancy stage should be considered when assessing vitamin D status. Nutritionally relevant cholecalciferol supplementation further increased vitamin D status. These data are relevant to the development of fortification and supplementation policies in pregnant women in West Africa.

## Introduction

Vitamin D is of relevance to maternal, fetal, and infant health globally in relation to pregnancy-related complications (e.g., pre-eclampsia and gestational diabetes), preterm birth, and infant-related outcomes (1, 2). In particular, meta-analyses of observational data (3, 4) and vitamin D supplementation trials (2, 5, 6) provide evidence for a beneficial effect of vitamin D on birth weight and size and reduced risk of being born small for gestational age. In relation to other health effects, although some data provide evidence for a beneficial effect of higher vitamin D status, these observations have yet to be consistently borne out in supplementation trials, e.g., in relation to gestational diabetes (7). Nevertheless, vitamin D deficiency in pregnancy remains a concern and is of interest to international organizations with the goal to optimize vitamin D status for maternal and infant health (8, 9).

Globally, 54% of pregnant women are estimated to have a plasma/serum total 25-hydroxyvitamin [25(OH)D] concentration <50 nmol/L and 18% <25 nmol/L indicating a concerning degree of vitamin D insufficiency and deficiency (10). More recent reports have detailed vitamin D status in pregnant women in Nigeria (11) and Tunisia (12), and indicated a 25(OH)D serum concentration of <50 nmol/L in 29% and 87%, respectively. However, it is evident that there is an uneven global distribution of studies, with few in African and Southeast Asian countries (13, 14). Authors of a recent report concluded that 65% of low- and middle-income countries had no published data that were suitable for inclusion in their systematic literature review (15). Furthermore, reports of vitamin D status from African countries are often of small sample size and have often used nonstandardized methods to measure vitamin D status. Therefore, there is a need for better characterization of vitamin D status in African regions particularly in pregnant women. Ethnic differences in vitamin D status and vitamin D metabolism [e.g., related to vitamin D binding protein (DBP) genotype, or parathyroid responsiveness] may also potentially impact vitamin D requirements and response to vitamin D supplementation in pregnancy, thus studies in different ethnic populations are warranted and are of importance in development of global nutrition policies (16).

Changes in 25(OH)D concentration, the primary marker of vitamin D status, are not well characterized throughout pregnancy and data are conflicting (17–24). Lack of longitudinal data, pregnancy-related physiological changes (e.g., changes in plasma volume and protein concentrations), and seasonal fluctuations lead to uncertainty over how vitamin D status may change in pregnancy and consequently whether thresholds for vitamin D deficiency are the same for nonpregnant and pregnant adults. Such targets are important in the context of not only ensuring vitamin D sufficiency in the mother, but also that of the newborn (24).

Pregnancy-related changes in other vitamin D metabolites, and whether they track changes in 25(OH)D, are not well defined (24, 25). Changes in 24,25-dihydroxyvitamin D [24,25(OH)_2_D] may provide information on the relative activation of catabolic pathways (26, 27), including in pregnancy where CYP24A1 may be downregulated (28). Changes in C3-epimer of 25-hydroxyvitamin D [3-epi-25(OH)D], a metabolite that may contribute to vitamin D activity, are also not certain. The reported contribution of either metabolite to vitamin D status will depend on the analytical methodology. Chromatographic methods that do not resolve 3-epi-25(OH)D from 25(OH)D and immunoassay methods for 25(OH)D that cross-react with 24,25(OH)_2_D may lead to an overestimation of vitamin D status (26, 29). In addition, some immunoassays may underreport 25(OH)D because of the presence of high DBP concentrations, as observed during pregnancy (30).

Vitamin D supplementation is generally effective in raising plasma/serum 25(OH)D concentrations, including in pregnant women (2, 24). However, there are few studies of vitamin D supplementation at doses (≤10 μg/d) in line with recommendations [e.g., from the Scientific Advisory Committee on Nutrition (31) or the Institute of Medicine estimated average requirement (32)] that may be more typical of population or nationwide supplementation or fortification programs (9). In pregnant women with known vitamin D deficiency, the WHO recommended nutrient intake is 5 μg/d (200 IU) (33).

Therefore, in a secondary analysis of samples collected as part of a randomized controlled trial of multiple-micronutrient supplementation including vitamin D, and designed to enhance infant immune development (34, 35), 25(OH)D and other vitamin D metabolites were quantified by LC-MS/MS. Samples were available from both early pregnancy (presupplementation) and late pregnancy. The aims of this work were to *1*) characterize vitamin D status in a large pregnancy cohort in sub-Saharan Africa, *2*) determine the impact on vitamin D status of nutritionally relevant daily doses of supplemental vitamin D (10 μg/d), and *3*) describe vitamin D metabolite concentrations across gestation.

## Methods

Samples and data were collected as part of the ENID (Early Nutrition and Infant Immune Development) trial (ISRCTN49285450) conducted from MRC Keneba, MRC Unit The Gambia in the rural area of West Kiang, a primarily subsistence farming community. At a latitude of 13°North, UVB-containing sunshine is available year-round (15), and most members of this predominantly Muslim community do not wear clothes that prevent sunshine exposure to hands, arms, and face. The Gambia has 2 distinct seasons, a “dry” season between November and May characterized by hot, sunny days, and a “wet” season between June and October with more cloud cover, higher humidity, and heavy rainfall. During the wet season, farming activities are at their peak. A majority of women undertake farming activities (36) and women continue these throughout pregnancy (37). Further details about the region and its demographics have been reported (38).

### Study information

Full details of the ENID trial have been published (34, 35, 39) and are summarized here in the context of the presented work. Women aged 18–45 y, not severely anemic (hemoglobin <7 g/dL) or with HIV infection, and premenopausal were recruited. The study was conducted according to the guidelines laid down in the Declaration of Helsinki and all procedures involving the participants were conducted as approved by the joint Gambia Government-MRC Ethics Committee (Project number SCC1126v2). Trained staff explained the study to participants, and informed, written consent was obtained.

Women were visited and interviewed monthly by fieldworkers. Pregnancy was initially indicated by human chorionic gonadotrophin (hCG) testing (QuickVue™ One-Step hCG urine test, bioMerieux) and then confirmed and gestational age assessed by ultrasound (Siemens ACUSON Antares Ultrasound Imaging System, Siemens Medical Solutions USA, Inc.). After confirmation of pregnancy, women were randomly assigned to 1 of the 4 intervention arms, iron-folic acid (FeFol) tablets, multiple micronutrient (MMN) tablets, a protein-energy (PE) lipid-nutrient supplement (LNS), or the PE supplement with multiple micronutrients (PE + MMN). FeFol is the standard supplement advised in pregnancy according to Gambian Government guidelines. MMN arms included 10 μg/d (400 IU) of cholecalciferol (vitamin D_3_). A full description of the contents of each supplement can be found in Moore et al. (34). Compliance percentage was determined either through counting the remaining tablets or estimation of remaining LNS supplement at the end of each week (39). Participants were included in this analysis if a plasma sample was available for vitamin D analysis either at recruitment (“early pregnancy” and presupplementation) or at 30 weeks of gestation (“late pregnancy”). Samples were collected throughout the year between February 2010 and October 2013.

### Data and sample collection

Participant data and anthropometry measurements were collected by trained fieldworkers or midwives, and are described in full elsewhere (34). Maternal blood samples were collected into lithium heparin blood tubes from a forearm vein in the morning after an overnight fast. Plasma was separated by centrifugation at at 1800 × *g* for 10 min at 4°C, stored at −70°C, and subsequently transported to the MRC Elsie Widdowson Laboratory on dry ice and stored at −70°C.

### Sample analysis

The quantitation of plasma 25-hydroxyergocalciferol [25(OH)D_2_], 25-hydroxycholecalciferol [25(OH)D_3_], and C3-epimer of 25-hydroxycholecalciferol [3-epi-25(OH)D_3_] was performed based on a published LC-MS/MS method (40) with modifications and the inclusion of 24,25-dihydroxycholecalciferol [24,25(OH)_2_D_3_] using a Waters Acquity ultra-performance liquid chromatography instrument and AB Sciex 5500 QTrap mass spectrometer. The method used separate isotope-labeled internal standards for each compound (for full details see **Supplemental Methods**). The limit of quantification (LOQ) was 1.5 nmol/L for 25(OH)D_2_, 25(OH)D_3_, and 3-epi-25(OH)D_3_ analytes, and 2.5 nmol/L for 24,25(OH)_2_D_3_. The MRC Elsie Widdowson Laboratory is a member of the Vitamin D Standardization Program, and quality assurance of the assay was performed as part of the Vitamin D External Quality Assessment Scheme (www.deqas.org) and performance assessed against NIST SRM 972a (for assay performance see **Supplemental Table 1**).

### Data analysis

Data analysis was performed with Stata 14.2 (StataCorp LLC). Because only a few samples contained 25(OH)D_2_ above the LOQ, all data analysis was performed with 25(OH)D_3_ only. Where vitamin D metabolite concentrations were less than the LOQ, values were computed by dividing the LOQ by the square root of 2 (41); assigned values were 1.1 nmol/L and 1.8 nmol/L for 3-epi-25(OH)D_3_ and 24,25(OH)_2_D_3_, respectively. Normally distributed data are presented as mean ± SD. Skewed data [i.e., 24,25(OH)_2_D_3_ and 3-epi-25(OH)D_3_ and their ratios] are presented as geometric mean and geometric mean SD. Parity is presented as median and range.

Supplement group differences in both early and late pregnancy, and differences between early and late pregnancy were tested by ANOVA. Pairwise comparison of group means was tested post hoc with Scheffé procedure. For skewed data, analysis was performed using logged values. Pearson's chi-square test was used for categorical data and the Kruskal-Wallis H to test for equality of medians (i.e., parity). Tests were not adjusted for weeks of pregnancy because there was no difference between supplement groups in this parameter.

The effect of season on 25(OH)D_3_ concentration was investigated with the use of Fourier regression (42, 43), with pairs of sine and cosine terms as independent predictors of 25(OH)D_3_. Annual data were aggregated by the day of year on which the blood sample was collected. The coefficient of cyclic variation summarized the magnitude of the seasonal variation (42). In multivariate regression models in early pregnancy, the sine and cosine terms were included to allow other covariates to be interpreted independent of seasonal effects.

We investigated biologically plausible predictors of early pregnancy 25(OH)D_3_ concentration with use of linear regression. Significant predictors of age and weeks of pregnancy were included in the multivariate model, which also included Fourier terms to control for any effect of time of year. To investigate predictors of attained 25(OH)D_3_, both early pregnancy 25(OH)D_3_ concentration and supplementation group were included in all models.

Vitamin D metabolite concentrations and their ratios were compared between pregnancy stage and supplementation group using ANOVA with Scheffé post hoc test. Percent 24,25(OH)_2_D_3_ and % 3-epi-25(OH)D_3_ refer to the relative concentration of the metabolite to 25(OH)D_3_ concentration, that is 24,25(OH)_2_D_3_/25(OH)D_3_ *100.

The effect of gestational age on vitamin D metabolite concentrations and the ratios of the metabolites to 25(OH)D were investigated using a mixed linear model including weeks of pregnancy (fixed effect) and participant ID (random effect). To describe the normal physiological pattern in pregnancy, this analysis was performed in the FeFol group only. Possible nonlinear associations were assessed by the inclusion of a quadratic term (predictor*predictor), but removed from the model if not significant (*P* > 0.05). Metabolite concentrations below the LOQ were excluded for this and for the following analysis; results with these data are included in the Supplemental Material (**Supplemental Table 2**).

Relations between vitamin D metabolites and their ratios against 25(OH)D_3_ concentration in early and late pregnancy were investigated using linear regression. Quadratic terms were also tested but were nonsignificant (*P* > 0.05). Data from the 4 groups were pooled because there were no supplement group differences in 25(OH)D and metabolite relations or ratios (investigated by the inclusion of an interaction term between supplement group and the predictor variable). Differences between early and late pregnancy in metabolite relations were tested with the inclusion of interaction term between time point (early/late pregnancy) and the continuous predictor variable.

## Results

### Presupplementation, early pregnancy participant characteristics, and vitamin D status

Participant characteristics and early pregnancy plasma 25(OH)D_3_ and vitamin D metabolite concentrations are shown in **Table 1**. There were no differences between supplement groups in age, weeks of pregnancy, anthropometric indices, or vitamin D metabolite concentrations (*P* > 0.5). In early pregnancy, the gestational age ranged between 7.0 and 20.8 wk. The distribution of the month of sampling was not different between supplementation groups (*P* = 1.0).

**TABLE 1:** Baseline characteristics and vitamin D status in early and late pregnancy for all participants and by supplement group^1^

	Early pregnancy						Late pregnancy				
		Supplement group^2^					Supplement group^2^				
	Full cohort	FeFol	MMN	PE	PE + MMN	*P*	FeFol	MMN	PE	PE + MMN	*P*
*n*	863	214	215	217	217		201	208	195	204	
Age, y	29.6 ± 6.7	29.9 ± 6.5	29.3 ± 6.7	29.1 ± 6.4	30.1 ± 7.0	NS	—	—	—	—	—
Weight, kg	55.6 ± 9.7	55.1 ± 9.0	55.5 ± 9.9	56.1 ± 9.3	55.8 ± 10.7	NS	—	—	—	—	—
Height, cm	161.8 ± 5.8	161.7 ± 6.1	162.0 ± 5.7	161.9 ± 5.6	161.6 ± 5.9	NS	—	—	—	—	—
BMI, kg/m^2^	21.2 ± 3.5	21.1 ± 3.2	21.1 ± 3.8	21.4 ± 3.3	21.3 ± 3.6	NS	—	—	—	—	—
MUAC,^3^ cm	26.7 ± 3.3	26.6 ± 3.1	26.7 ± 3.6	26.8 ± 3.0	26.7 ± 3.4	NS	—	—	—	—	—
Triceps skinfold thickness,^3^ cm	14.7 ± 6.4	14.9 ± 7.1	14.3 ± 6.0	14.9 ± 6.2	14.8 ± 6.2	NS	—	—	—	—	—
Nulliparous,^4^*n* (%)	71 (8.3)	17 (8.0)	21 (9.8)	19 (8.8)	14 (6.5)	NS	—	—	—	—	—
Parity,^5^[median (range)]	4 (0–12)	4 (0–12)	4 (0–10)	4 (0–11)	4 (0–11)	NS	—	—	—	—	—
Weeks of pregnancy^6^	13.7 ± 3.3	13.8 ± 3.4	13.7 ± 3.3	13.7 ± 3.3	13.5 ± 3.1	NS	—	—	—	—	—
Plasma 25(OH)D_3_, nmol/L	70.2 ± 15.3	70.0 ± 15.7	71.4 ± 15.4	70.5 ± 14.7	69.1 ± 15.3	NS	81.5 ± 18.2*	94.9 ± 18.8*^a^	87.0 ± 17.2*^b^	90.3 ± 20.4*^a,b^	<0.0001
∆25(OH)D_3_^7^	—	—	—	—	—	—	10.8 ± 14.0	23.4 ± 14.9^a^	15.7 ± 14.0	21.2 ± 15.6^a^	<0.0001
25(OH)D_3_ <50 nmol/L,^8^*n* (%)	64 (7.4)	23 (10.7)	12 (5.6)	12 (5.5)	17 (7.8)	NS	8 (4.0)*	0 (0.0)*	3 (1.5)*	3 (1.5)*	0.02
25(OH)D_3_ ≥125 nmol/L, *n* (%)	2 (0.2)	1 (0.5)	1 (0.5)	0 (0)	0 (0)	NS	3 (1.5)	16 (7.7)*	4 (2.1)*	15 (7.4)*	0.002

1 Data are means ± SDs unless otherwise indicated. *P* value is for difference between groups [ANOVA with Scheffé test, chi-square or Kruskal-Wallis H to test for equality of medians (for parity)]. ^a,b,c^Labeled means in a row without a common superscript letter differ, *P* < 0.05. *Indicates within supplement group significant difference from early pregnancy for continuous variables. FeFol, iron folic acid; MMN, multiple micronutrients; MUAC, midupper arm circumference; PE, protein energy; PE + MMN, protein energy with multiple micronutrients; NS, nonsignificant (*P* ≥ 0.05); 25(OH)D_3_, 25-hydroxycholecalciferol.

2 MMN and PE + MMN supplements include 10 µg/d cholecalciferol.

3 Observations were not available for all participants, *n* = 858 (full cohort), *n* = 212 (FeFol), *n* = 213 (MMN), *n* = 216 (PE).

4 Observations were not available for all participants, *n* = 859 (full cohort), *n* = 213 (FeFol), *n* = 215 (MMN), *n* = 215 (PE), *n* = 216 (PE + MMN).

5 Values are median (range). Observations were not available for all participants, *n* = 846 (full cohort), *n* = 210 (FeFol), *n* = 212 (MMN), *n* = 213 (PE), *n* = 211 (PE + MMN).

6 Observations were not available for all participants, *n* = 862 (full cohort), *n* = 213 (FeFol).

7 Observations were not available for all participants, *n* = 200 (FeFol), *n* = 206 (MMN), *n* = 195 (PE), *n* = 204 (PE + MMN).

8 No participants had a 25(OH)D_3_ concentration <30 nmol/L.

In early pregnancy, the mean plasma 25(OH)D_3_ concentration was 70.2 ± 15.3 nmol/L. No woman had 25(OH)D_3_ <30 nmol/L. 25(OH)D_2_ was present above the LOQ in 50 (6%) samples in early pregnancy and in these women the mean concentration was 1.8 ± 0.4 nmol/L, consistent with low availability of vitamin D_2_ in this population.

### Attained 25(OH)D_3_ concentration in late pregnancy

**FIGURE 1:**
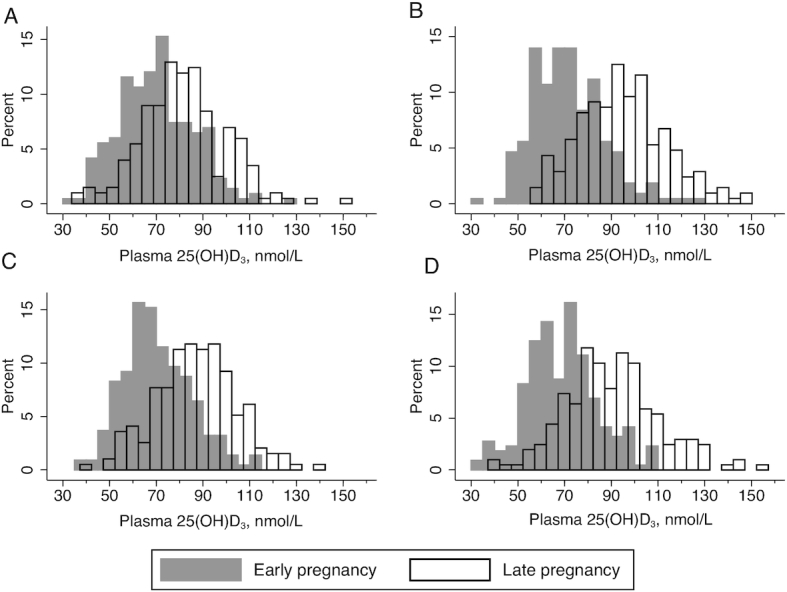
Distribution of plasma 25(OH)D_3_ concentrations by pregnancy stage and supplement group. (A) FeFol; (B) MMN; (C) PE; (D) PE + MMN. FeFol, iron folic acid; MMN, multiple micronutrients; PE, protein energy; PE + MMN, protein energy with multiple micronutrients; 25(OH)D_3_, 25-hydroxycholecalciferol.

By late pregnancy, vitamin D status had significantly increased in each of the 4 supplementation groups, that is in those receiving no vitamin D and those receiving micronutrient supplements containing vitamin D (Table 1), with the shift to significantly higher concentration distribution of 25(OH)D_3_ concentration clearly visible in the vitamin D-supplemented groups (**Figure 1**). Attained 25(OH)D_3_ concentration was significantly higher in the two vitamin D-containing supplementation groups and in the PE group compared to the FeFol group (Table 1), but there was no difference between the MMN and PE + MMN groups.

Expressed as the difference between early and late pregnancy, the change in 25(OH)D_3_ concentration was not different between MMN-containing groups nor between the non-MMN containing groups (i.e., FeFol and PE groups) (Table 1). In the FeFol group, the change was +10.8 ± 14.0 nmol/L and 20% of participants had a decrease in 25(OH)D_3_ concentration. In contrast, in the MMN only group, 5% of participants had a decrease in 25(OH)D_3_ concentration between early and late pregnancy. Vitamin D supplementation shifted the cohort distribution, reducing the percentage of participants in the MMN group with 25(OH)D_3_ concentration <50 nmol/L to zero, but increasing the proportion with a concentration >125 nmol/L (Figure 1; Table 1).

### Predictors of early pregnancy 25(OH)D_3_ concentration

**FIGURE 2:**
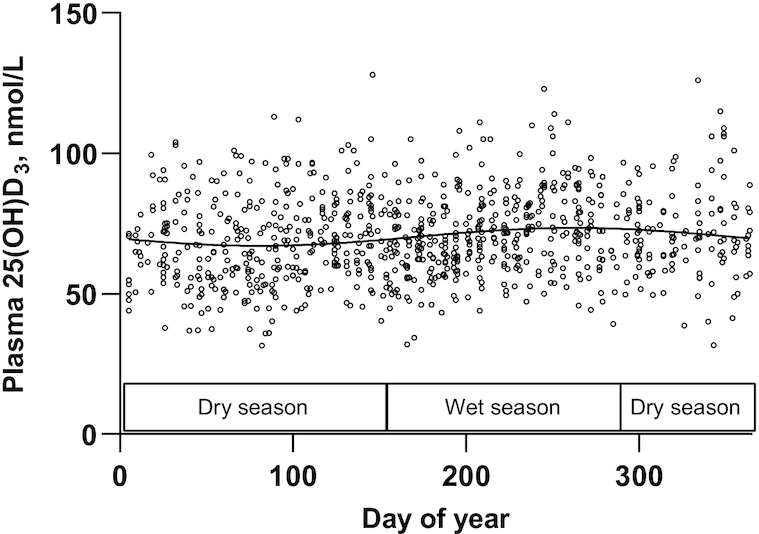
Seasonal variation in plasma 25(OH)D_3_ concentration in early pregnancy. Observed (open circles) and Fourier regression modeled seasonal variation (solid line) in plasma 25(OH)D_3_ concentration in 862 women measured in early pregnancy. Day 1 constitutes 1 January in each year. Boxes indicate dry (November to May) or wet season (late June to early October). 25(OH)D_3_, 25-hydroxycholecalciferol.

**FIGURE 3:**
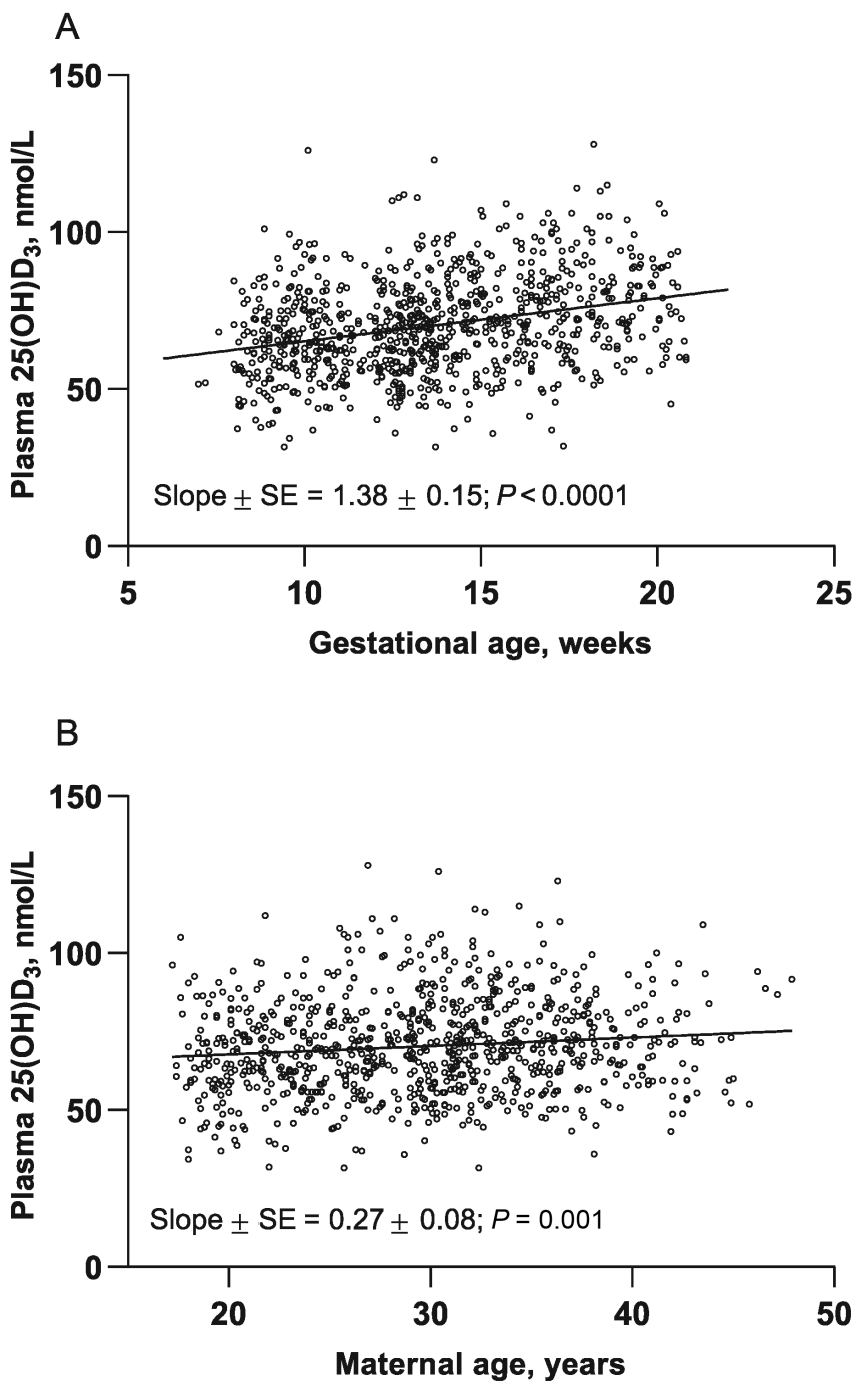
The relations between (A) weeks of pregnancy and (B) maternal chronological age and plasma 25(OH)D_3_ concentration in early pregnancy. Open symbols are observed 25(OH)D_3_ concentration. Solid line is the modeled line. 25(OH)D_3_, 25-hydroxycholecalciferol.

There was a small but significant effect of time of year on 25(OH)D_3_ plasma concentration (**Figure 2**). The difference between the modeled peak and nadir 25(OH)D_3_ concentration was 6.4 nmol/L, with the peak in September and nadir in March. The SD of this seasonal component was 2.2 nmol/L (coefficient of cyclic variation = 4.4%) (after correction for age, weight, and weeks of pregnancy). Investigated predictors of early pregnancy, presupplementation, and 25(OH)D_3_ concentration are shown in **Table 2**. 25(OH)D_3_ concentration was not associated with maternal size or adiposity, but maternal age and weeks of pregnancy positively predicted 25(OH)D_3_ concentration (**Figure 3**). A multivariate model including age, weeks of pregnancy, and Fourier terms to control for the effect of time of year explained 11% of the variability in plasma 25(OH)D_3_, with weeks of pregnancy predicting a 1.34 nmol/L higher 25(OH)D_3_ concentration for a 1-wk increase in gestational stage (*P* < 0.0001) (Table 2).

**TABLE 2:** Predictors of early pregnancy plasma 25(OH)D_3_ concentration^1^

		Univariate model		Multivariate model	
	*n*	β ± SE	*P*	β ± SE	*P*
Age, y	863	0.27 ± 0.08	0.001	0.18 ± 0.07	0.015
Weight, kg	863	−0.03 ± 0.05	0.6	—	—
Height, cm	863	−0.02 ± 0.09	0.8	—	—
BMI, kg/m^2^	863	−0.06 ± 0.15	0.7	—	—
MUAC, cm	858	0.01 ± 0.16	1.0	—	—
Triceps skinfold thickness, cm	858	−0.04 ± 0.08	0.6	—	—
Weeks of pregnancy	862	1.38 ± 0.15	<0.0001	1.34 ± 0.15	<0.0001

1 Biologically plausible predictors of 25(OH)D_3_ concentration were tested and the multivariate model included Fourier terms to control for the effect of the time of year. Factors were measured on the same day as blood sample collection. Parity was not included in the multivariate model because of a strong correlation with age. MUAC, midupper arm circumference; 25(OH)D_3_, 25-hydroxycholecalciferol.

### Participant characteristics as predictors of attained 25(OH)D

Participant characteristics influencing 25(OH)D_3_ concentration in late pregnancy were further investigated using a combined model with presupplementation, baseline 25(OH)D_3_ concentration, and study arm as covariates. In these combined models there were no significant interactions between predictor variables and supplementation group. Attained 25(OH)D_3_ concentration was positively associated with compliance and weeks on supplementation, and negatively with late pregnancy triceps skinfold thickness (**Table 3**). For every 1 cm greater triceps skinfold thickness in late pregnancy, attained 25(OH)D_3_ concentration was 0.2 nmol/L lower (*P* = 0.02). There were no associations with other anthropometric indices measured in either early or late pregnancy.

**TABLE 3:** Predictors of attained plasma 25(OH)D_3_ concentration in late pregnancy^1^

		Univariate model^2^		Multivariate model^2^, ^3^	
	*n*	β ± SE	*P*	β ± SE	*P*
Age, y	803	0.07 ± 0.08	0.4		
Gestational age in late pregnancy, wk	803	−0.77 ± 0.56	0.2	−0.62 ± 0.55	0.3
Weight in early pregnancy, kg	803	−0.06 ± 0.05	0.3	—	—
Weight in late pregnancy, kg	802	−0.06 ± 0.05	0.3	—	—
Change in weight between early and late pregnancy, kg	802	−0.03 ± 0.17	0.8	—	—
MUAC in early pregnancy, cm	802	−0.14 ± 0.16	0.4	—	—
MUAC in late pregnancy, cm	803	−0.25 ± 0.16	0.1	—	—
Triceps skinfold thickness in early pregnancy, cm	802	−0.11 ± 0.0	0.2	—	—
Triceps skinfold thickness in late pregnancy, cm	803	−0.21 ± 0.09	0.02	−0.21 ± 0.09	0.02
Compliance, %	803	0.10 ± 0.04	0.02	0.10 ± 0.04	0.01
Weeks on supplement	798	0.97 ± 0.15	<0.0001	0.98 ± 0.15	<0.0001

1 MUAC, midupper arm circumference; 25(OH)D_3_, 25-hydroxycholecalciferol.

2 Early 25(OH)D_3_ concentration and supplementation group were included as covariates. Interactions between predictor variables and supplementation group were nonsignificant.

3 *n* = 798.

### 25(OH)D_3_ and vitamin D metabolites across gestation

**FIGURE 4:**
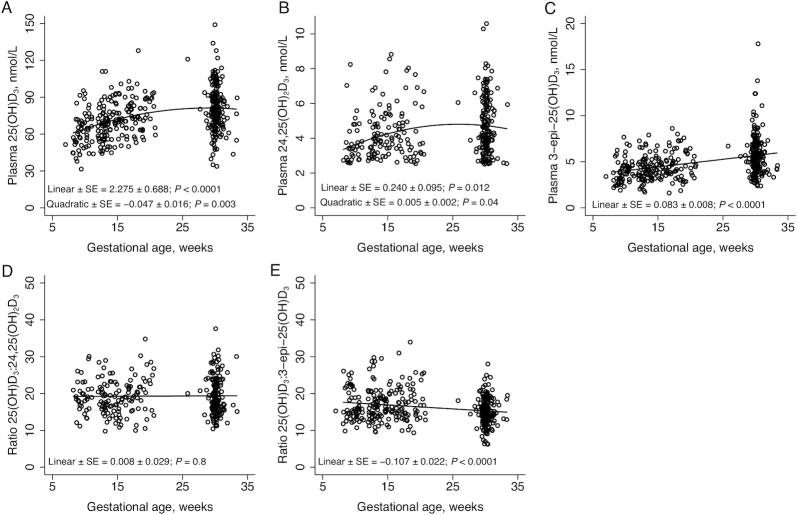
Relations between plasma 25(OH)D_3_ and vitamin D metabolites with gestational age determined with mixed model linear regression with random effect of participant ID and fixed effect of gestational age. Linear and quadratic terms ± SE are indicated on the graphs. (A) plasma 25(OH)D_3_, *n* = 215; (B) plasma 24,25(OH)_2_D_3_, *n* = 183; (C) plasma 3-epi-25(OH)D_3_, *n* = 215; (D) ratio of plasma 25(OH)D_3_ to 24,25(OH)_2_D_3_, *n* = 183; (E) ratio of plasma 25(OH)D_3_ to 3-epi-25(OH)D_3_, *n* = 215. 3-epi-25(OH)D_3_, C3-epimer-25-hydroxycholecalciferol; 24,25(OH)_2_D_3_, 24,25-dihydroxycholecalciferol; 25(OH)D_3_, 25-hydroxycholecalciferol.

We observed quadratic relations in both 25(OH)D_3_ and 24,25(OH)_2_D_3_ across gestation such that the positive linear increase observed in early pregnancy appeared to flatten towards late pregnancy. In contrast, 3-epi-25(OH)D_3_ concentration increased as pregnancy progressed (**Figure 4**A, B, C). These observations were also reflected in metabolite ratios against weeks of pregnancy (Figure 4D, E); the 25(OH)D_3_:24,25(OH)_2_D_3_ ratio was consistent across gestational age, whereas we observed a significant negative relation between the 25(OH)D_3_:3-epi-25(OH)D_3_ ratio and gestational age.

### Vitamin D metabolites in early and late pregnancy

**FIGURE 5:**
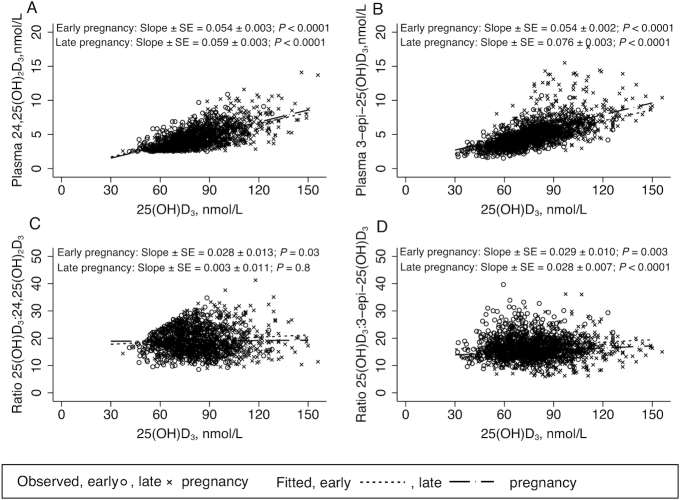
Relations in early and late pregnancy between 25(OH)D_3_ concentration and (A) plasma 24,25(OH)_2_D_3_ (early pregnancy: *n* = 580, late pregnancy: *n* = 721); (B) plasma 3-epi-25(OH)D_3_ (early: *n* = 839, late: *n* = 791); (C) ratio of plasma 25(OH)D_3_ to 24,25(OH)_2_D_3_ (early: *n* = 580, late: *n* = 721); (D) ratio of plasma 25(OH)D_3_ to 3-epi-25(OH)D_3_ (early: *n* = 839, late: *n* = 791). 3-epi-25(OH)D_3_, C3-epimer-25-hydroxycholecalciferol; 24,25(OH)_2_D_3_, 24,25-dihydroxycholecalciferol; 25(OH)D_3_, 25-hydroxycholecalciferol.

Plasma concentrations of 24,25(OH)_2_D_3_ are reported in **Table 4**. There was a positive linear relation between 24,25(OH)_2_D_3_ and 25(OH)D_3_ concentration that was not different between early and late pregnancy (*P* = 0.3 interaction) (**Figure 5**A). In early pregnancy, there was a slight positive linear relation between 25(OH)D_3_:24,25(OH)_2_D_3_ and 25(OH)D_3_ (*P* = 0.03), but this was not significantly different from the nonsignificant relation observed in late pregnancy (*P* = 0.1 for interaction) (Figure 5C). We also performed the same regression analyses using assigned values for 24,25(OH)_2_D_3_ concentrations that were below the LOQ (Supplemental Table 2). In this scenario, slopes between 24,25(OH)_2_D_3_ and 25(OH)D_3_ were similar to those obtained using data above the LOQ. However, in contrast to using data above the LOQ, there were significant negative linear relations between the ratio 25(OH)D_3_:24,25(OH)_2_D_3_ with 25(OH)D_3_ in both early (β ± SE) (−0.082 ± 0.019, *P* < 0.0001) and late pregnancy (−0.060 ± 0.013, *P* < 0.0001).

**TABLE 4:** Early and late pregnancy plasma vitamin D metabolites by supplement group^1^

	Early pregnancy						Late pregnancy				
		Supplement group^2^					Supplement group^2^				
	Total cohort	FeFol	MMN	PE	PE + MMN	*P*	FeFol	MMN	PE	PE + MMN	*P*
24,25(OH)_2_D_3_											
*n*	778	190	199	196	193		196	203	184	194	
24,25(OH)_2_D_3_, nmol/L^§^	3.3 ± 1.5	3.3 ± 1.6	3.3 ± 1.5	3.1 ± 1.5	3.4 ± 1.6	NS	4.0 ± 1.6*^a^	4.4 ± 1.5*^a,b^	4.8 ± 1.4*^b,c^	5.0 ± 1.5*^c^	<0.0001
*n* (%) below LOQ	198 (25)	48 (25)	45 (23)	56 (29)	49 (25)	—	26 (13)	14 (7)	7 (4)	5 (9)	—
∆24,25(OH)_2_D_3_,^3^ nmol/L	—	—	—	—	—	—	0.9 ± 1.7^a^	1.3 ± 1.4^a^	1.8 ± 1.6^b^	2.0 ± 2.0^b^	<0.0001
%24,25(OH)_2_D_3_:25(OH)D_3_^§^	4.7 ± 1.4	4.7 ± 1.5	4.6 ± 1.5	4.5 ± 1.5	4.9 ± 1.6	NS	5.0 ± 1.4^a^	4.7 ± 1.3^a^	5.5 ± 1.3*^b^	5.7 ± 1.4*^b^	<0.0001
25(OH)D_3_:24,25(OH)D_2_D_3_ ratio^§^	21.4 ± 1.4	21.2 ± 1.5	21.6 ± 1.5	22.3 ± 1.5	20.6 ± 1.6	NS	20.0 ± 1.4^a^	21.4 ± 1.3^a^	18.0 ± 1.3*^b^	17.7 ± 1.4*^b^	<0.0001
3-epi 25(OH)D_3_											
* n*	842	207	209	214	212	—	201	206	185	199	—
3-epi 25(OH)D_3_, nmol/L^§^	4.1 ± 1.4	4.1 ± 1.4	4.2 ± 1.3	4.0 ± 1.4	4.0 ± 1.4	NS	5.4 ± 1.4*^a^	6.3 ± 1.4*	5.7 ± 1.4*^a^	5.7 ± 1.4*^a^	0.0001
*n* (%) below LOQ	3 (0.4)	1 (0.5)	0 (0)	2 (0.9)	0 (0)	—	0 (0)	0 (0)	0 (0)	0 (0)	—
∆3-epi-25(OH)D_3_,^4^ nmol/L	—	—	—	—	—	—	1.5 ± 2.2^a^	2.2 ± 2.2^b^	1.8 ± 1.9^a,b^	1.8 ± 2.1^a,b^	0.007
%3-epi-25(OH)D_3_:25(OH)D_3_^§^	6.0 ± 1.3	6.0 ± 1.3	6.1 ± 1.3	5.8 ± 1.3	6.0 ± 1.3	NS	6.8 ± 1.3*	6.7 ± 1.3*	6.7 ± 1.3*	6.5 ± 1.3*	0.4
25(OH)D_3_:3-epi 25(OH)D_3_ ratio^§^	16.7 ± 1.3	16.6 ± 1.3	16.5 ± 1.3	17.1 ± 1.3	16.6 ± 1.3	NS	14.7 ± 1.3*	14.8 ± 1.3*	15.0 ± 1.3*	15.4 ± 1.3*	0.4

1 Data are means ± SDs or geometric means ± geometric mean SDs^§^. *P* value is for differences between groups (ANOVA with Scheffé test). ^a,b,c^Labeled means in a row without a common superscript letter differ, *P* < 0.05. *Indicates within supplement group significant difference from early pregnancy for continuous variables. FeFol, iron folic acid; LOQ, limit of quantitation; MMN, multiple micronutrients; PE, protein energy; PE + MMN, protein energy with multiple micronutrients; 3-epi-25(OH)D_3_, C3-epimer-25-hydroxycholecalciferol; 24,25(OH)_2_D_3_, 24,25-dihydroxycholecalciferol; 25(OH)D_3_, 25-hydroxycholecalciferol.

2 MMN and PE + MMN supplements include 10 µg/d cholecalciferol.

3 Observations were not available for all participants, *n* = 195 (FeFol), *n* = 201 (MMN), *n* = 184 (PE), *n* = 193 (PE + MMN) .

4 Observations were not available for all participants, *n* = 200 (FeFol), *n* = 204 (MMN), *n* = 184 (PE), *n* = 198 (PE + MMN).

Plasma concentrations of 3-epi-25(OH)D_3_ are reported in Table 4. In late pregnancy, the mean concentration and mean %3-epi-25(OH)D_3_ were higher than in early pregnancy across all supplementation groups (Table 4). The slopes of the significant positive linear relations between 25(OH)D_3_ and both 3-epi-25(OH)D_3_ concentration and their ratios were not different between early and late pregnancy (*P* > 0.4) (Figure 5B and D).

## Discussion

We have characterized vitamin D status, response to supplementation, and vitamin D metabolite concentrations in a large cohort of pregnant women resident in rural Gambia. This study generates important findings in relation to vitamin D physiology in pregnancy. Firstly, we observed that during pregnancy in women with relatively constant vitamin D supply from cutaneous synthesis and generally good vitamin D status, 25(OH)D_3_ concentration increased by 11 nmol/L across pregnancy in the group not receiving supplemental vitamin D. Weeks of pregnancy was a strong predictor of vitamin D status and should therefore be considered in the interpretation of studies of vitamin D status in pregnancy. Secondly, in groups receiving 10 μg/d (400 IU/d) vitamin D_3_ within a micronutrient supplement, there was an additional ∼11 nmol/L increase in 25(OH)D_3_ concentration across pregnancy. Thirdly, in women not receiving vitamin D supplementation, 3-epi-25(OH)D_3_ concentration continued to increase across pregnancy suggesting an independent effect of pregnancy on production of 3-epi-25(OH)D_3_.

This is the largest study of vitamin D status in a pregnancy cohort in Africa. The Gambia lies relatively close to the equator at 13°N and therefore receives year round UVB-containing sunshine (15). Although we detected some seasonality in vitamin D status, which followed predictions of UVB availability, the effect was relatively modest and was equal to a difference of ∼6 nmol/L between the peak and nadir. To put this in context, it is comparable to the magnitude of diurnal variation we have observed in elderly people in The Gambia, the UK, and China (43), and is considerably lower than seasonal variation observed in pregnant women at higher latitudes (22, 44).

Vitamin D status was relatively high in this population with a mean of ∼70 nmol/L in early pregnancy; there was no vitamin D deficiency [25(OH)D concentration <30 nmol/L] (31, 32). This proportion is lower than some reports in pregnant women in Africa (11, 12) and probably reflects the relatively high UVB availability, skin exposure, and outdoor lifestyle in this rural Gambian population (38). The majority of circulating 25(OH)D is derived from exposure to sunlight, including in The Gambia, and the population has little or no access to fortified foods. Mean 25(OH)D concentration at baseline was similar to other cohorts from regions of similar latitude and supports the notion of a physiological norm described previously (45). Although no single population could be used to conclude what is normal, and other local environmental factors and related metabolic adaptations may need to be considered, this cohort provides a unique data set that could be considered as representative of the physiological norm for vitamin D metabolism in pregnancy.

Weeks of pregnancy was a strong positive predictor of 25(OH)D_3_ concentration in early pregnancy and indicates that this variable should be accurately determined and included in the analysis of studies of vitamin D status during pregnancy. The observed ∼11 nmol/L increase in 25(OH)D_3_ concentration across pregnancy is equivalent to the increase expected with an oral intake of ∼400 IU/d of vitamin D. Reported changes in vitamin D status with gestation are inconsistent, partly because of the large seasonal influence present in many studies, with suggestions of increases (20, 46), (47), decreases (23), and no change over pregnancy (17, 48). However, mean differences can disguise large interindividual variation (17). The physiological basis for the observed increase in 25(OH)D concentration may relate to an increase in DBP during pregnancy. The change we observed in 25(OH)D_3_ concentration up to ∼32 weeks of pregnancy closely mirrors that observed in other studies for DBP with an increase in early pregnancy followed by flattening between ∼28 and 32 weeks of pregnancy (23). This may imply that under circumstances where vitamin D supply from UVB exposure is relatively unrestricted, as in The Gambia, plasma 25(OH)D_3_ responds to an increase in DBP concentration. Such a response cannot occur where vitamin D supply is low or seasonal. This may indicate that during pregnancy, when DBP increases, the % free 25(OH)D is maintained, when permitted by a sufficiently high supply of vitamin D. Recent data from a study in pregnant US adolescents suggested a slight negative association between % free 25(OH)D and 25(OH)D concentration, although free 25(OH)D was highly correlated with 25(OH)D and did better predict associations with parathyroid hormone (49). In early pregnancy, we also observed a significant positive association between maternal age and 25(OH)D_3_ concentration. A similar, albeit smaller effect, was also observed in a UK pregnancy cohort measured in late pregnancy (50). In The Gambia, this association may be related to secular trends in style of dress or time spent outside engaged in farming, gardening, or other activities.

Vitamin D supplementation in this largely vitamin D-replete population had a positive effect on vitamin D status and reduced the proportion of women with both 25(OH)D_3_ concentration <50 nmol/L and the proportion with a decrease in vitamin D status between early and late pregnancy. The observed change in 25(OH)D_3_ concentration with daily doses of 10 μg/d was consistent with the dose-response observed in studies in other ethnic groups, including in pregnancy (2, 24, 51). An advantage of this study was that we were able to separate and demonstrate increases resulting from pregnancy per se from those associated with supplementation. A similar pregnancy-independent increase was also observed in a recent vitamin D supplementation trial in Ireland; however, seasonal changes in UVB supply were also influential (24), whereas our observations are largely independent of season. We demonstrate the positive effect on vitamin D status of a nutritionally relevant vitamin D supplement dose that is in line with amounts typically used in supplementation programs or that are obtainable through fortification. These data are relevant to the debate around the design of vitamin D supplementation trials and ethical concerns over the use of true placebo or active placebo (where the control group consists of a “low” dose supplementation) as the comparative arm, as commonly applied in randomized controlled trials of pharmacological agents (52, 53).

Dietary reference values for pregnant women are typically the same or based on an incremental increase over the recommendations for nonpregnant adults (9, 24). The reasons for this are attributable to the lack of data on pregnancy-related health outcomes, uncertainty over whether there is a metabolic increase in vitamin D requirements, and, until recently (24), a lack of data on the dose-response in pregnancy. The WHO recommendation is that sunlight exposure is the most important source of vitamin D and that the evidence does not support the use of supplemental vitamin D in pregnancy, with the exception of where there is confirmed vitamin D deficiency (33). Normal physiological changes in vitamin D status in pregnancy have been difficult to quantify partly because of the seasonal variation in vitamin D supply exhibited in Europe and the United States, where the majority of pregnancy studies have been performed. More recent studies have used seasonal correction to model 25(OH)D change in pregnancy, but data remain conflicting on the gestational-related change in 25(OH)D concentration (19, 22). In a study using stable isotope-labeled 25(OH)D, we have previously shown that 25(OH)D expenditure was not different between pregnant and nonpregnant women (21). Together with the data from the current study and based on the study of vitamin D status, this suggests that in this population, metabolically, pregnant women may not require more vitamin D than nonpregnant women and that metabolic adaptations or efficiencies are able to maintain vitamin D status where vitamin D supply is adequate and constant. Further confirmatory studies in other populations with for example, different vitamin D status, different DBP polymorphisms, or levels of adiposity, are required. However, this finding does not remove the need to ensure that all pregnant women are vitamin D sufficient. As discussed above, recent meta-analyses provide evidence for a beneficial effect of vitamin D on birth weight and size. Furthermore, recent data suggest that higher vitamin D intakes for pregnant women may be needed to ensure a plasma/serum 25(OH)D_3_ concentration in cord blood of >50 nmol/L (24).

Vitamin D supplementation in our study increased the proportion of women with 25(OH)D_3_ concentration >125 nmol/L, the lower end of the range (125–150 nmol/L) at which the Institute of Medicine reported as a cause for concern (while also recognizing the lack of data) (32). As reviewed elsewhere (2), maternal supplementation studies report few adverse effects (e.g., cases of hypercalcemia) (51, 54–56). However, these reports in pregnancy do not negate the concerns around higher vitamin D doses in relation to increased risks of falls and fractures nor with individuals within a population with medical disorders that may predispose to hypercalcemia (31), and the effects on fetal development are unknown.

Evidence suggests a negative association between response to vitamin D supplementation and measures of adiposity in both pregnant and nonpregnant women (50, 57). We did not find a relation between vitamin D status and BMI. This may be explained by the relatively low and narrow range of BMI observed in this population. Furthermore, as discussed below, assessment of adiposity during pregnancy is challenging. In our study, we found a negative association between late-pregnancy triceps skinfold thickness and 25(OH)D_3_ concentration, after adjustment for other factors. The relation with skinfold thickness may only have become apparent in late pregnancy after oral vitamin D because of differences in vitamin D transport. The tissue distribution of oral vitamin D that is initially transported by chylomicrons and lipoproteins may be different to that of cutaneously synthesized vitamin D transported by DBP (57). Population body composition may need to be considered when designing supplementation programs in different populations. Changes in body fat mass are more challenging to measure in pregnancy than in nonpregnant adults. However, the use of triceps skinfold thickness has been found to be a good estimate of fat mass in the absence of more invasive, advanced methods (58). We observed large interindividual variation in change of 25(OH)D_3_ concentration over pregnancy and in response to supplementation (2). The factors that influence this variation remain to be elucidated but may relate to differences in metabolism (because of genetic polymorphisms, other dietary or hormonal factors, or differences in lifestyle). We did not measure dietary calcium intake in this cohort. However, studies in this population indicate that calcium intakes are generally low (<400 mg/d) by international standards and do not change in pregnancy (21). Other studies in The Gambia (59) and in an Irish population (60) indicated that vitamin D use was not affected by low calcium intake.

In early pregnancy, vitamin D metabolites, 24,25(OH)_2_D_3_ and 3-epi-25(OH)D_3_, increased with rising 25(OH)D_3_ concentration. For 24,25(OH)_2_D_3_, the increase was followed by a flattening that closely mirrored that of 25(OH)D_3_. In contrast, 3-epi-25(OH)D_3_ concentration continued to rise, an observation that was reflected when its ratio was plotted against weeks of pregnancy. This is consistent with recent work that reported an increase in 3-epi-25(OH)D_3_ as pregnancy progressed (24, 61). As a percentage of 25(OH)D_3_ concentration, the 3-epi-25(OH)D_3_ results are similar to those estimated from the results from some studies (24) but higher than others (61). The source and biological significance of the relatively higher 3-epi-25(OH)D_3_ concentration in pregnancy, as well as in neonates (61), are unknown (62). It is suggested that production of 24,25(OH)_2_D_3_ may be suppressed at lower 25(OH)D_3_ concentrations (<∼25 nmol/L) (63), and Best et al. reported that 24,25(OH)_2_D_3_ concentration in pregnancy was related to both gestational stage and 25(OH)D_3_ concentration (25). Our results suggest that 24,25(OH)_2_D_3_ may be more related to 25(OH)D_3_ because the ratio across gestation did not change. The 24,25(OH)_2_D_3_:25(OH)D_3_ ratio is lower than reported in studies of nonpregnant adults (26), but consistent with a previous report of pregnant women. Lower relative 24,25(OH)_2_D may be related to a downregulation of the CY24A1 enzyme in pregnancy (24).

An advantage of this study was our ability to determine changes in vitamin D status across pregnancy in a relatively large number of individuals where the effect of season was small. In contrast to some studies, the use of an LC-MS/MS method that fully resolved and quantified 25(OH)D_2_, 25(OH)D_3_, 3-epi-25(OH)D_3_, and 24,25(OH)_2_D_3_ provides confidence over the change in vitamin D status over pregnancy as well as providing data on vitamin D metabolite changes over pregnancy.

This study was limited by factors related to the original study design. The latest sample collected during pregnancy was around 30 weeks of gestation, thus we are not able to infer changes in vitamin D status nearer delivery. We did not collect dietary data on vitamin D but vitamin D-containing foods or supplements are not considered to be significant sources of vitamin D in this tropical, rural, largely subsistence-farming community and thus the changes we observed over pregnancy can be interpreted in the context of a stable vitamin D supply from UVB-containing sunshine. We did not include variables related to income, education, or other social economic scores. However, other reports of this cohort report limited heterogeneity in these factors (64, 65). The limit of quantification of our LC-MS/MS method for 24,25(OH)_2_D_3_ was higher than in some other studies (25) and meant we were not able to include all participants in the 24,25(OH)_2_D_3_ models.

In conclusion, this study provides an accurate assessment of vitamin D status in a pregnancy cohort in rural Gambia and contributes to data on vitamin D status in low- and middle-income countries that are scarce and may be limited by sample size and the choice of vitamin D assay. Importantly, this study provides information on 25(OH)D and vitamin D metabolite response to pregnancy and vitamin D supplementation when vitamin D supply is stable from year-round high UVB availability. The generalizability of these findings to other populations requires further study in different groups. The potential relevance of nutritionally relevant doses (10 µg/d) and their impact in vitamin D supplementation trials and relevance to the development of vitamin D fortification and supplementation policies in pregnant women are highlighted.

## Supplementary Material

nxz290_Supplemental_File

## Notes

Supported by UK Medical Research Council program codes U105960371 and U123261351 (KSJ and AP and sample analysis), MC-A760-5QX00 and the UK Department for International Development (DFID) under the MRC/DFID Concordat agreement (ENID trial) and MC_UP_1005/1 (SEM). KSJ and SRM are supported by the National Institute for Health Research (NIHR) Cambridge Biomedical Research Centre (BRC) (IS-BRC-1215-20014). The NIHR Cambridge BRC is a partnership between Cambridge University Hospitals NHS Foundation Trust and the University of Cambridge, funded by NIHR. The views are those of the authors and not necessarily those of the NHS, the NIHR, or the UK Department of Health and Social Care.

Author disclosures: The authors report no conflicts of interest.

Supplemental Methods and Supplemental Tables 1 and 2 are available from the “Supplementary data” link in the online posting of the article and from the same link in the online table of contents at https://academic.oup.com/jn.

Abbreviations used: DBP, vitamin D binding protein; FeFol, iron and folic acid supplement; LOQ, limit of quantification; MMN, multiple micronutrients; PE, protein energy; PE + MMN, protein energy supplement with multiple micronutrients; 1,25(OH)_2_D, 1,25-dihydroxyvitamin D; 3-epi-25(OH)D_3_, C3-epimer of 25-hydroxycholecalciferol; 24,25(OH)_2_D_3_, 24,25-dihydroxycholecalciferol; 25(OH)D_3_, 25-hydroxycholecalciferol.
