# Supplementary material for: Vitamin D Status Increases During Pregnancy and in Response to Vitamin D Supplementation in Rural Gambian Women
Source: J Nutr. 2019 Dec 13;150(3):492–504. doi: 10.1093/jn/nxz290 (PMC7056615; doi:10.1093/jn/nxz290)
Supplement: nxz290_Supplemental_File [file nxz290_supplemental_file.docx]

**Online Supplementary Material**

**Vitamin D status increases during pregnancy and in response to vitamin D supplementation in rural Gambian women**

**Kerry S. Jones, Sarah R. Meadows, Inez Schoenmakers, Ann Prentice, Sophie E. Moore**

**Supplementary methods**

**Sample preparation**

200 µL of sample or QC material was mixed with 200 µL methanol and 150 µL IS solution to precipitate proteins. Liquid extraction was performed with hexane (1.5 mL) for 9 minutes and then centrifuged. After centrifugation the full hexane layer was removed into a 96-well 2 mL polypropylene plate and dried down under vacuum before being reconstituted in 200 µL 73% methanol.

**Calibrators and reagents**

Calibrators were prepared from 25(OH)D_2_, 25(OH)D_3_, 3-epi-25(OH)D_3_ and 24,25(OH)_2_D_3_ and hexadeuterated 25(OH)D_3_ and 24,25(OH)_2_D_3_ and trideuterated 25(OH)D_2_ and 3-epi-25(OH)D_3_ were used as internal standards (IS) (Sigma-Aldrich, Dorset, UK). All compounds were dissolved separately in ethanol and stored at -70^°^C. The exact concentration of the stock solutions was calculated using absorbance at 264 nm and molar absorption coefficients. Working standards were made in 73% methanol to give ranges of 2.5 – 100 nmol/L for 25(OH)D_2_, 5-150 nmol/L for 25(OH)D_3_, 1.67 – 50 nmol/L for 3-epi-25(OH)D_3_ and 0.625 – 20 nmol/L for 24,25(OH)_2_D_3_. IS were prepared in 73% methanol to give concentrations of 50 nmol/L d3 25(OH)D_2_, 100 nmol/L d6 25(OH)D_3_, 25 nmol/L d3 3-epi-25(OH)D_3_ and d6 24,25(OH)_2_D_3_. Quality control (QC) material was in-house pooled plasma from donor samples, MassCheck 3-*epi*-25-OH-vitamin D_3_/D_2_ and 25-OH-vitamin D_3_/D_2_ Serum Control Levels I and II (Chromsystems, Grafelfing, Germany). Standards were diluted with the IS at a ratio of 4:3 before injection onto the UPLC.

**LC-MS/MS analysis**

The LC-MS/MS system consisted of a Waters Acquity UPLC and AB Sciex 5500 QTrap mass spectrometer fitted with an atmospheric pressure chemical ionization (APCI) probe. Calibrators and samples were injected (37.5 µL) onto a reversed-phase column (Thermo Scientific Hypersil GOLD PFP 2.1 x 100mm 1.9 µm fully porous particles with an in-line filter). Chromatographic separation was performed under isocratic conditions using 73% methanol at a flow rate of 350 µL/min and column temperature of 30°C for 8 minutes followed by a column wash with 100% methanol for 3 minutes. The MS was operated in positive ionization mode using the transitions 25(OH)D_3_ 383.3>365.3/257.3; 25(OH)D_2_ 395.4>377.3/209.2; 3-epi-25(OH)D_3_ 383.4>365.3/257.2; 24,25(OH)D_2_D_3_ 399.4>381.3/363.4; d6-25(OH)D_3_ 389.4>371.3; d3-25(OH)D_2_ 398.4>380.4; d3-3-epi-25(OH)D_3_ 404.4>386.3; 24,25(OH)D_2_D_3_ 405.4>387.3. Analyte peak area to IS peak area ratio was compared to that of a calibration curve to determine analyte concentration.

The inter-assay variation, calculated using 10 replicates of either QC material or spiked serum was <10% for all analytes and the limit of quantification (LOQ), calculated using 10 x SD of repeat measurements of a low sample, was 1.5 nmol/L for 25(OH)D_2_, 25(OH)D_3_ and 3-epi-25(OH)D_3_ analytes and 2.5 nmol/L for 24,25(OH)_2_D_3_. The limit of detection (LOD) was 1.1 nmol/L for all compounds. The MRC Elsie Widdowson Laboratory was a member of the Vitamin D Standardization Program (VDSP), and quality assurance of the assay was performed as part of the Vitamin D External Quality Assessment Scheme (www. deqas.org).

**Supplementary Table 1. NIST SRM 972a and DEQAS performance data**

Supplementary Table 1 contains a summary of performance data for NIST SRM 972a and DEQAS during the period of assay work up and analysis on the AB Sciex 5500 QTrap mass spectrometer. Data are only reported where the target concentration is above the LOQ for the assay and consequently the sample number for analytes ranges from 2 to 4 for NIST 972a and 6 to 20 for DEQAS. Target concentrations are obtained from NIST or DEQAS and based on reference measurement procedures except for 24,25(OH)_2_D_3_ that is the mean of the nine DEQAS participating laboratories that provided data.

|  |  | % difference | | | nmol/L difference | | |
| --- | --- | --- | --- | --- | --- | --- | --- |
|  | *n* | Mean | Min | Max | Mean | Min | Max |
| **NIST 972a** |  |  |  |  |  |  |  |
| 25(OH)D_2_ | 2 | 2.9 | -5.0 | 10.8 | 2.9 | -5.0 | 10.8 |
| 25(OH)D_3_ | 8 | -0.5 | -8.7 | 6.0 | -0.5 | -3.9 | 4.4 |
| 3-epi-25(OH)D_3_ | 4 | -12.9 | -19.6 | -1.9 | -2.6 | -7.6 | -0.8 |
| 24,25(OH)_2_D_3_ | 4 | -14.3 | -21.1 | -3.8 | -0.7 | -1.0 | -0.2 |
| **DEQAS** |  |  |  |  |  |  |  |
| 25(OH)D_2_ | 6 | -9.6 | -21.1 | 0.5 | -0.2 | -0.4 | 0.1 |
| 25(OH)D_3_ | 20 | -2.3 | -9.1 | 8.5 | -2.0 | -8.1 | 6.0 |
| 3-epi-25(OH)D_3_ | 7 | -3.5 | -25.3 | 38.1 | -0.8 | -2.2 | 0.9 |
| 24,25(OH)_2_D_3_ | 15 | -4.1 | -41.4 | 95.0 | -0.5 | -3.6 | 3.8 |

**Supplementary Table 2. Regression coefficients for relationships between vitamin D metabolites in early and late pregnancy.**

Analysis presented in the manuscript excluded values below the LOQ (Figure 5). Supplementary Table 2 presents these relationships with the inclusion of assigned values where 24,25(OH)_2_D_3_ or 3-epi-25(OH)D_3_ were below the LOQ.

|  |  | **Early pregnancy** | | | **Late pregnancy** | | | |
| --- | --- | --- | --- | --- | --- | --- | --- | --- |
| y-variable | x-variable | *n* | Slope ± SE) | *P* | *n* | Slope ± SE) | *P* | *P* for interaction between early and late pregnancy |
|  |  |  |  |  |  |  |  |  |
| Plasma 24,25(OH)_2_D_3_, nmol/L | 25(OH)D_3_ | 778 | 0.066 ± 0.003 | <0.0001 | 777 | 0.065 ± 0.003 | <0.0001 | 0.9 |
| Plasma 3-epi-25(OH)D_3_, nmol/L | 25(OH)D_3_ | 842 | 0.055 ± 0.002 | <0.0001 | 791 | 0.058 ± 0.003 | <0.0001 | 0.5 |
| Ratio 24,25(OH)_2_D_3_: 25(OH)D_3_ | 25(OH)D_3_ | 778 | -0.082 ± 0.019 | <0.0001 | 777 | -0.060 ± 0.013 | <0.0001 | 0.3 |
| Ratio 3-epi-25(OH)D_3_: 25(OH)D_3_ | 25(OH)D_3_ | 842 | 0.020 ± 0.010 | 0.04 | 791 | 0.028 ± 0.007 | <0.0001 | 0.5 |
